# A Route to Synthesize Ionizable Lipid ALC-0315, a Key Component of the mRNA Vaccine Lipid Matrix

**DOI:** 10.1134/S1068162023020061

**Published:** 2023-05-19

**Authors:** I. A. Boldyrev, V. P. Shendrikov, A. G. Vostrova, E. L. Vodovozova

**Affiliations:** 1 Shemyakin–Ovchinnikov Institute of Bioorganic Chemistry, Russian Academy of Sciences, 117997 Moscow, Russia; 2 Mendeleev University of Chemical Technology of Russia, 125047 Moscow, Russia

**Keywords:** ionizable lipid, ALC-0315, synthesis, lipid nanoparticles, mRNA

## Abstract

The ionizable lipid ALC-0315, ((4-hydroxybutyl)azanediyl)bis(hexane-6,1-diyl)bis(2-hexyldecanoate), is a component of the lipid matrix of the prophylactic SARS-CoV-2 mRNA vaccine produced by Pfizer/BioNTech. This lipid ensures efficient vaccine assembly, protects the mRNA from premature degradation, and promotes the release of the nucleic acid into the cytoplasm for its further processing after endocytosis. The present work describes a simple and economical method for the synthesis of the ALC-0315 lipid, which can be taken advantage of in mRNA vaccine production.

## INTRODUCTION

The SARS-CoV-2 coronavirus pandemic which broke out late in 2019 promoted intensive development, rapid marketing and introduction into clinical practice of highly efficient prophylactic vaccines based on lipid nanoparticles (LNP) and the full-length virus spike protein (S-protein) mRNA produced by Moderna [1, 2] and Pfizer/BioNTech [3]. These vaccine constructs are supramolecular systems, specifically, nanoparticles consisting of multiple lipid molecules and mRNA molecule(s) (for example, see the reviews [4, 5]). The structural and functional roles of lipid molecules consist in facilitating compact packaging of long-chain nucleic acid molecules, protecting them from degradation by extracellular enzymes, providing for the nanoparticle transport into the cell via endocytosis (phagocytosis or pinocytosis) and subsequent mRNA release from the endosome (phagosome) into the cytoplasm for translation and expression of the immunogenic epitope-containing protein. Presumably, mRNA entry into the cytoplasm is a result of LNP-constituting lipids transition to their cationic form under the acidic conditions in the endosome, which promotes their contact with anionic lipids in the periplasmic monolayer of the endosome membrane and further membrane destabilization due to the formation of nonbilayer lipid structures (hexagonal phase) [6]. Taking into account the diversity of functions, the lipid platform of mRNA-containing LNPs should include several classes of lipids. The key component is ionizable lipids, which ensure both mRNA packaging during LNP assembly and mRNA release into the cytoplasm. The structures of ionizable lipids in the vaccines produced by both companies are very close, and the protocols for their synthesis are described in the corresponding patents [7–9].

**Fig. 1.**
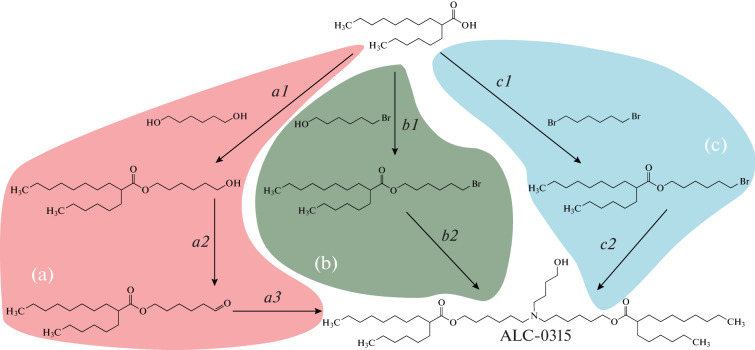
Routes of synthesis of the ALC-0315 lipid.

The commercial success of mRNA vaccines based on ionizable lipids led to an explosive growth of interest in the developments in this field and, as a consequence, to the search for simplified methods for the synthesis of lipids with proven efficiency, in particular, the ALC-0315 lipid, ((4-hydroxybutyl)azanediyl)bis(hexane-6,1-diyl)bis(2-hexyldecanoate). Two ways of this lipid synthesis starting from 2-hexyldecanoic acid are currently known (Figs. 1a, 1b). The alternative variant proposed in this work is shown in Fig. 1c.

## RESULTS AND DISCUSSION

The first route of ALC-0315 synthesis (Fig. 1a) is covered by a patent [8, 9]. Step *a1* is the acylation of an excess of hexanediol under the standard Steglich esterification conditions [10] (condensation with dicyclohexylcarbodiimide in the presence of 4-dimethylaminopyridine as a catalyst). The reaction product is further used without purification. Step *a2* is the oxidation with pyridinium chlorochromate; the product is extracted with hexane or petroleum ether, separated by chromatography on a silica gel column in the hexane–methylene chloride system. The final step *a3* is the reductive amination. Sodium triacetoxyborohydride is the key reagent in this reaction; the reagent is commercially available or can be obtained *in situ* in the reaction between sodium borohydride and glacial acetic acid in benzene or dimethylacetamide [11]. The target product ALC-0315 is obtained by chromatography on a silica gel column using a methanol gradient in methylene chloride. The described synthesis route has two drawbacks: (1) oxidation and reduction of the end carbon atom (steps *a2* and *a3*) are used consecutively resulting in the oxidation state of the carbon atom remaining the same as it was initially (+1), which indicates that oxidation and reduction may possibly be substituted by condensation; (2) a side product, an urea derivative, is produced at the first stage (step *a1*), i.e., Steglich esterification, which complicates the purification of the end product.

An alternative synthesis route (Fig. 1b) has been recently proposed in a patent [12]. In this scheme, the oxidation–reduction problem was solved by using bromohexanol instead of hexanediol. This allowed us to reduce the number of stages. However, the esterification in the presence of a water-reducing agent remained.

We propose further improvement of the ALC-0315 synthesis scheme (Fig. 1c) by using dibromohexane at the first stage. The simplicity of purification and the possibility to recycle dibromohexane, along with the use of K_2_CO_3_ as a condensing agent instead of the more expensive and hazardous carbodiimide derivatives make this synthesis route economically attractive.

We synthesised the ALC-0315 lipid in two steps (Fig. 1c). 2-Hexyldecanoic acid was reacted with a fourfold excess of 1,6-dibromohexane in DMF in the presence of potash. After three days of stirring the reaction mixture at room temperature, the condensation reaction product was isolated by chromatography on silica gel with 85% yield. Dibromohexane recovery rate was 52%. The target product ALC-0315 was obtained in the condensation reaction between 2-hexyldecanoic acid, 6-bromohexyl ester and 4-aminobutanol under the same mild conditions as in the previous step. With only a 10% excess of 6-bromohexyl-2-hexyl decanoate according to the stoichiometric ratio of the reagents, the yield of the target product after silica gel chromatography separation was 62%. In addition, the mixture of the target product with the mono-alkylation product was obtained, which was added to 6-bromohexyl-2-hexyl decanoate during subsequent repeats of synthesis. Taking into account recycling and silica gel column regeneration (consecutive washing with isopropanol and chloroform) the yield of the target product reached 80%. The structure of the obtained target compound was confirmed by the ^1^H-NMR spectra.

**Fig. 2.**
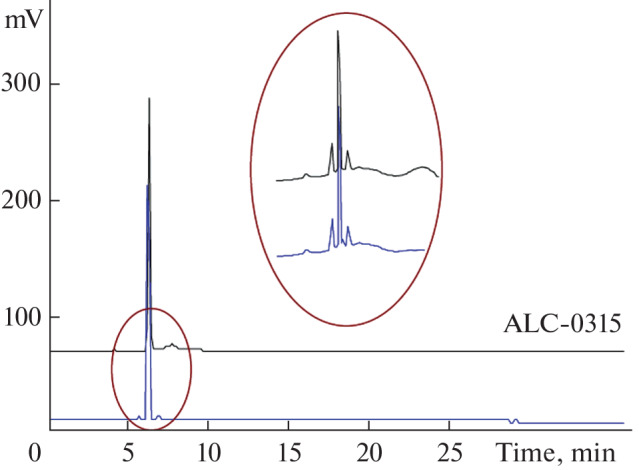
Chromatograms of the obtained ionizable lipid (blue line) and the ALC-0315 standard (black line). HPLC conditions are provided in the “Experimental.”

The purity of the product was monitored by HPLC. The chromatograms show higher purity level of the synthesized target compound compared to the commercial ALC-0315 product (Fig. 2).

## EXPERIMENTAL

Decanoic acid, hexyl bromide, 1,6-dibromohexane, 4-aminobutanol (Reachim, Russia); butyllithium (Acros Organics, United States); diisopropylamine, 4-dimethylaminopyridine (Merck, Germany); sodium sulfate, hexane, ethyl acetate, tetrahydrofuran, chloroform, methylene chloride, methanol, glacial acetic acid (Ltd TC KHIMMED, Russia). The solvents were purified according to standard procedures. Solutions were concentrated in a rotary evaporator (Heidolph, Germany). Vacuum drying using an oil pump was performed in the Inei-4 lyophilic dryer (Institute of Biological Instrumentation, Russian Academy of Sciences, Pushchino, Russia).

2-Hexyldecanoic acid was obtained as described in Ramishetti et al. [13]. Thin-layer chromatography (TCL) was performed on the Kieselgel 60 plates (Merck, Germany); for compound detection, plates were sprayed with Rhodamine-6G solution in ethanol (0.16 mM) with orange spots appearing on a pink background under the UV lamp. KSCG 0.063–0.2 mm silica gel (Ltd ChromLab, Russia) was used for column chromatography. Gas chromatography was performed on a Crystallux gas chromatograph (Ltd Metachrom, Russia), using Zebron-ZB-1 capillary column (Phenomenex, United States). HPLC was performed using the JETchrom chromatographic system (PortLab, Russia) consisting of two pumps and a dynamic mixer, the Rheodyne 7125 injector (IDEX, United States) and the Sedex-LC LT-ELSD light scattering detector (Sedere, France). Chromatographic data were collected and processed using the MultiChrom 3.4 software (Ltd Ampersend, Russia). ^1^H-NMR spectra were recorded on the Avance 700 spectrometer (Bruker, United States) with the operating frequency of 700 MHz at the constant sample temperature of 303 K. СDCl_3_ was used as the solvent. The chemical shifts in the NMR spectra are provided in parts per million (ppm); residual solvent proton signals (7.27 ppm) were used as the internal standard.

**2-Hexyldecanoic acid, 6-bromohexyl ester.** In a 50‑mL round bottom flask, 9.74 g (4 equiv, 39.9 mmol) of 1,6-dibromohexane was mixed with 2.56 g (1 equiv, 9.98 mmol) of 2-hexyl decanoic acid and 20 mL of dry DMF. Then, 2.05 g of K_2_CO_3_ (chemically pure; potash was first ground in a mortar and dried at 100°C for 30 min) was added. The mixture was stirred for 72 h, and the formation of the colourless fine KBr precipitate was observed. The course of reaction was monitored using TLC in the petroleum ether–ethyl acetate–acetic acid, 39 : 1 : 0.05, system. *R*_f_ for 1,6-dibromohexane, the target compound, hexane diether and 2-hexyl decanoic acid were 0.98, 0.59, 0.38 and 0.11, respectively. To the reaction mixture, 200 mL of water were added, stirred thoroughly and extracted with ethyl acetate (3 × 100 mL). Combined extracts were washed with water, dried over Na_2_SO_4_ and evaporated in a rotary evaporator. The residue was applied on a silica gel column and separated using a stepwise gradient, namely, the initial dibromohexane was eluted with petroleum ether and the product was obtained using the petroleum ether–ethyl acetate system (3 : 1). The yield was 3.58 g (85%), in the form of a colourless transparent liquid, with 5.08 g of dibromohexane being regenerated.

^1^H NMR spectrum (CDCl_3_ , δ, ppm; SSIC—*J*, Hz): 4.15 (t, *J* 6.7, 2H), 3.47 (t, *J* 6.8, 2H), 2.42–2.35 (br. m, 1H), 1.91 (quint, *J* 7.0, 2H), 1.71 (quint, *J* 7.0, 2H), 1.69–1.61 (m, 2H), 1.58–1.43 (m, 6H), 1.40–1.27 (br. m, 20H) and 0.95 (two overlapping t, *J* 7.2, 6H).

**4-Hydroxybutyl-bis(6-(2-hexyldecanoate)hexyl)-amine (systematic name [(4-hydroxybutyl)aza-nediyl]di(hexane-6,1-diyl)bis(2-hexyl decanoate)).** In a 50-mL round bottom flask, 3 g (7.15 mmol) of 6-bromohexyl-2-hexyl decanoate, 0.286 g (3.21 mmol) of 4-butanolamine, 1 g of K_2_CO_3_ (prepared as described above) and 20 mL of dry DMF were combined. The mixture was stirred for 72 h. The course of reaction was monitored by TLC in the chloroform-methanol-triethylamine system, 95 : 5 : 0.1. *R*_f_ for the target product and mono-alkylation product were 0.35 and 0.1, respectively. Reaction mixture was treated as described above for 6-bromohexyl-2-hexyl decanoate. The residue was applied on a silica gel column, and the chloroform–methanol, 97 : 3, system was used. The yield of the pure target product was 1.54 g (62%). The next fraction was collected as containing the mixture of mono- and di-alkylation products of 4-butanolamine (1.6 g), which were used in the repeated synthesis.

HPLC of the target product and the ALC-0315 standard (BroadPharm, United States) was performed on the C18 Gravity column, 150 × 4.6 mm, 5 μm (Macherey-Nagel GmbH & Co. KG, Germany) in the following gradient: eluent A, 0.1% TFA in H_2_O; eluent B, methanol; flow rate 1.2 mL/min.

^1^H NMR spectrum (CDCl_3_, δ, ppm; SSIC*—J*, Hz): 4.12 (t, *J* 6.7, 4H), 3.62 (br. t, *J* 5.5, 2H), 2.53–2.47 (br. m, 4H), 2.40–2.34 (m, 2H), 1.75–1.60 (m, 12H), 1.59–1.52 (m, 4H), 1.52–1.41 (m, 10H), 1.39–1.27 (br. m, 46H) and 0.94 (two overlapping t, *J* 7.2, 12H).

## CONCLUSIONS

A new method for the ionizable ALC-0315 lipid production was suggested, which is distinctive for its simplicity and economical use of reagents. Taking into account the growing interest in mRNA vaccines based on lipid nanoparticles we believe that this method may be helpful in the manufacturing of this kind of vaccine.

## References

[1] Baden L.R., El Sahly H.M., Essink B., Kotloff K., Frey S., Novak R., Diemert D., Spector S.A., Rouphael N., Creech C.B., McGettigan J., Khetan S., Segall N., Solis J., Brosz A., Fierro C., Schwartz H., Neuzil K., Corey L., Gilbert P., Janes H., Follmann D., Marovich M.M.D., Mascola J., Polakowski L., Ledgerwood J., Graham B.S., Bennett H., Pajon R., Knightly C., Leav B., Deng W., Zhou H., Han S., Ivarsson M., Miller J., Zaks T. (2021). N. Engl. J. Med.

[2] Anderson E.J., Rouphael N.G., Widge A.T., Jackson L.A., Roberts P.C., Makhene M., Chappell J.D., Denison M.R., Stevens L.J., Pruijssers A.J., McDermott A.B., Flach B., Lin B.C., Doria-Rose N.A., O’Dell S., Schmidt S.D., Corbett K.S., Swanson P.A., Padilla M., Neuzil K.M., Bennett H., Leav B., Makowski M., Albert J., Cross K., Edara V.V., Floyd K., Suthar M.S., Martinez D.R., Baric R., Buchanan W., Luke C.J., Phadke V.K., Rostad C.A., Ledgerwood J.E., Graham B.S., Beigel J.H. (2020). N. Engl. J. Med.

[3] Polack F.P., Thomas S.J., Kitchin N., Absalon J., Gurtman A., Lockhart S., Pérez J.L., Perez Marc G., Moreira E.D., Zerbini C., Bailey R., Swanson K.A., Roychoudhury S., Koury K., Li P., Kalina W.V., Cooper D., Frenck R.W., Hammitt L.L., Tureci O., Nell H., Schaefer A., Ünal S., Tresnan D.B., Mather S., Dormitzer P.R., Şahin U., Jansen K.U., Gruber W.C. (2020). N. Engl. J. Med.

[4] Schoenmaker L., Witzigmann D., Kulkarni J.A., Verbeke R., Kersten G., Jiskoot W., Crommelin D.J.A. (2021). Int. J. Pharm.

[5] Hou X., Zaks T., Langer R., Dong Y. (2021). Nat. Rev. Mater.

[6] Szoka F.C., Xu Y., Zelphati O. (1997). Adv. Drug Delivery Rev.

[7] Benenato, K.E., Kumarasinghe, E.S., and Cornebise, M., US Patent Application no. 20170210697 A1, 2017.

[8] Ansell, S.M. and Du, X., US Patent no. 10166298 B2, 2019.

[9] Ansell, S.M. and Du, X., Int. Application WO no. 2017075531 A1, 2017.

[10] Neises B., Steglich W. (1978). Angew. Chem., Int. Ed. Engl.

[11] Gribble, G.W. and Abdel-Magid, A.F., Sodium triacetoxyborohydride, in Encyclopedia of Reagents for Organic Synthesis. https://onlinelibrary.wiley.com/doi/ abs/10.1002/9780470842898.rs112.pub2.

[12] Yan, D. and Xueming, T., CN Patent no. 114249662 A, 2022.

[13] Ramishetti S., Hazan-Halevy I., Palakuri R., Chatterjee S. (2020). Naidu, GonnaS., Dammes, N., Freilich, I., Kolik, ShmuelL., Danino, D., and Peer, D. Adv. Mater.

